# Descriptive Study of Foodborne Disease Using Case Monitoring Data in Shandong Province, China, 2016–2017

**Published:** 2019-04

**Authors:** Guangjian WU, Liansen WANG, Qiang WANG, Ru HAN, Jinshan ZHAO, Zunhua CHU, Maoqiang ZHUANG, Yingxiu ZHANG, Kebo WANG, Peirui XIAO, Ya LIU, Zhongjun DU

**Affiliations:** 1. School of Public Health, Jilin University, Changchun, China; 2. Shandong Center for Disease Control and Prevention, Jinan, China; 3. Shandong Center for Food Safety Risk Assessment, Jinan, China; 4. Academy of Preventive Medicine, Shandong University, Jinan, China; 5. Department of Preventive Medicine and Public Health Laboratory Science, School of Medicine, Jiangsu University, Zhenjiang, China; 6. Shandong Academy of Occupational Health and Occupational Medicine, Shandong Academy of Medical Sciences, Jinan, China

**Keywords:** Foodborne diseases, Case monitoring, Surveillance, Acute gastroenteritis, Assessment

## Abstract

**Background:**

In order to generate data on the burden of foodborne diseases in Shandong Province, we aimed to use the case monitoring data of foodborne diseases from 2016 to 2017 to estimate.

**Methods:**

Data were obtained from the foodborne disease surveillance reporting system with dates of onset from Jan 1, 2016, to Dec 31, 2017, in Shandong, China.

**Results:**

The places of food exposure were categorized by settings as follows: private home, catering facility, collective canteens, retail markets, rural banquets and other. Exposed food is divided into 23 categories. Overall incidence rate and proportions by exposure categories, age, and sex-specific incidence rates were calculated and sex proportions compared. Approximately 75.00% of cases who had at least one exposure settings were in private homes. The most frequently reported exposed food was a variety of food (meaning more than two kinds of food). The two-year average incidence rate was 75.78/100,000, sex-specific incidence rate was much higher for females compared to males (78.23 vs. 74.69 cases per 100,000 population). An age-specific trend was observed in the cases reported (Chi-Square for linear trend, χ^2^=4.39, *P*=0.036<0.05).

**Conclusion:**

A preliminary estimate of 14 million cases of foodborne diseases in Shandong province each year. Future studies should focus on cross-sectional and cohort studies to facilitate the assessment of the distribution and burden of foodborne disease of the population in Shandong. Considering strengthening the burden of foodborne diseases in foodborne disease surveillance is also a feasible way.

## Introduction

Both in developed and developing countries, foodborne diseases are becoming a greater challenge to public health (1). A large part of the world’s morbidity and mortality are caused by foodborne diseases although the exact mortality is unknown (2). Many countries have begun to undertake specific studies to understand the burden of diseases commonly transmitted by food very early, England and the Netherlands were among them, and the two countries have achieved some results. Domestically acquired foodborne illnesses resulted in 2.9 million cases in 1992 and 1.3 million cases in 2000 were estimated by investigators of England (3). The standardized number of acute gastroenteritis cases for Netherlands was 4.5 million (4). Although many countries have tried to study the burden of foodborne diseases, however, the real burden of foodborne diseases is not clear including China.

Many agents (e.g., a variety of bacteria, viruses, parasites, and chemicals) can cause food contaminated, and different pathogens and host factors (e.g. age and immunity) lead to different proportions of food transmission, only a small number of illnesses can be confirmed by laboratory testing and reported to public health agencies, thus lead to the assessment of the burden of food-borne diseases is very complex (5). Both the United States and Australia have adopted intensive disease monitoring methods to establish a project to accurately assess the population’s burden of diseases (FoodNet and OzFoodNet) (6). About ten years ago, America based on the FoodNet data, considering the uncertainty to estimate the burden of foodborne disease in the United States (5, 7). From 2001 to 2002, a cross-sectional telephone survey of acute gastroenteritis in the population was conducted in Australia for 12 months, estimating the foodborne proportion of acute gastroenteritis based on the data of each of the 16 pathogens in Australia (8). Global estimates of the full extent of the burden and cost of foodborne diseases are still unknown, although some countries have studied the burden of foodborne diseases, it is thought to be substantial. In order to generate data on the global burden of foodborne diseases, WHO launched an initiative in 2006 (9).

China carried out a large scale nationwide investigation of acute gastroenteritis in the 1980s. And China National Center for Food Safety Risk Assessment cooperated with Shanghai, Jiangsu, Zhejiang, Jiangxi, Guangxi, Sichuan Provincial Center for Disease Control and Prevention launched a cross-sectional survey on acute gastroenteritis in the population, from July 2010 to July 2011, however, Shandong has failed to participate in it.

The use of retrospective review is key to implementing effective food safety measures (10). Data statistical analysis is an effective tool for retrospective review, especially surveillance data are often recognized as one of the main evidence bases for the formulation of public health policies (9). In order to generate data on the burden of foodborne diseases in Shandong province, we are trying to use the case monitoring data of foodborne diseases from 2016 to 2017 to estimate.

## Methods

### Data sources

The case monitoring of foodborne diseases in Shandong Province began in 2013, and all cases are reported through the foodborne disease surveillance reporting system. At the beginning of 2016, sentinel hospitals of case monitoring of foodborne diseases, covering all secondary hospitals and tertiary hospitals in the province. Data for this study were obtained from the foodborne disease surveillance reporting system with dates of onset from Jan 1, 2016, to Dec 31, 2017. Surveillance case definition of foodborne diseases is an infectious or toxic medical case caused by food or suspected food (Cite from national foodborne disease monitoring manual in 2016 and in 2017, they were not allowed to disclose their contents without permission). Population data used for the calculation of incidence rates were obtained from Shandong Provincial Bureau of Statistics (11).

### Data Analysis Food exposure information

In the foodborne disease surveillance reporting system, the places of food exposure were categorized by settings as follows: private home, catering facility, collective canteens, retail markets, rural banquets and other. Catering facility including restaurant (hotel), food store, street food, and catering industry-the other. Collective canteens including unit canteen, school canteen and the canteen of construction sites. Food retail markets including farmers market, supermarket, retail store and retail-the other. Exposures that did not fit into these categories were categorized as “unclassifiable”. These exposures along with exposures reported as “unknown” and exposures that were missing were removed from analyses as appropriate.

### Categories of exposed food

In the foodborne disease surveillance reporting system, exposed food is divided into 23 categories, as follows: meat and meat products, vegetable and its products, fruit and its products (including preserved fruit and preserves), aquatic animals and their products, infant food, milk and dairy products, egg and egg products, drinks and frozen drinks, packaged drinking water (including barreled water), grain and its products (including starch sugar, roasting and all kinds of staple food), bean and bean products, nut seed and its products, fungi and their products, liquor and its products, candy, chocolate, honey and their products, algae and their products, oils & fats, condiment, health products, other foods, a variety of foods (meaning more than two kinds of food), mixed food and unidentified food.

### Incidence of foodborne disease

The incidence rate of foodborne disease in different regions and age groups were calculated. All data analysis was performed using Epi Info 7 (https://www.cdc.gov/epiinfo/support/downloads.html).

## Results

### Food exposure information

**Fig. 1:**
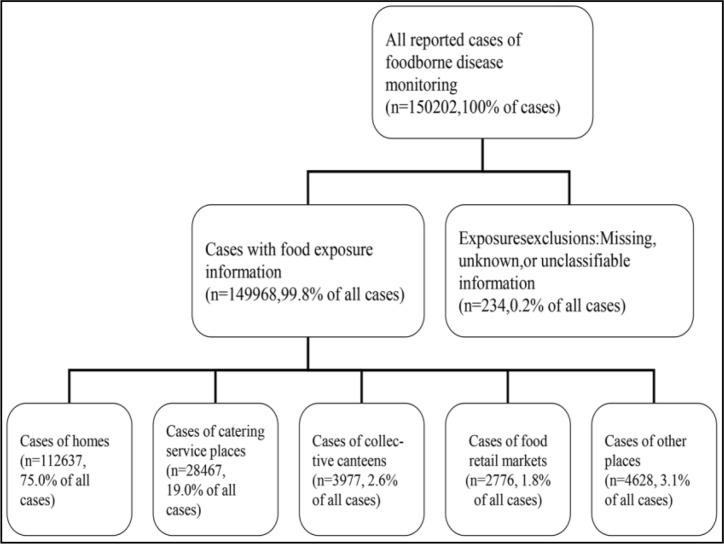
Number of surveillance cases of five reportable settings in each stage of selection for final analytic sample of cases, Shandong, 2016–2017

From Jan 1, 2016, to Dec 31, 2017, 150202 surveillance cases of foodborne diseases were reported in Shandong province, China (Fig. 1). Of these, 234 (0.2% of all cases) cases with missing, unknown or unclassifiable exposure information were removed, leaving 149968 cases that had at least one exposure settings reported.

Private home was the most frequently reported exposure setting. Overall, 112637 (75.0%) homes exposure cases were reported to have occurred among the 149968 cases. Furthermore, at least 1/3 of the 112637 cases (36044 cases) were purchased from a restaurant, a food store. Catering facility was the second frequently reported exposure setting with 28647 (19.0%) exposures, followed by other places with 4628 (3.1%), collective canteens with 3977 (2.6%), food retail markets with 2776 (1.8%), and rural banquets at 328 (0.2%).

### Categories of exposed food

**Fig. 2:**
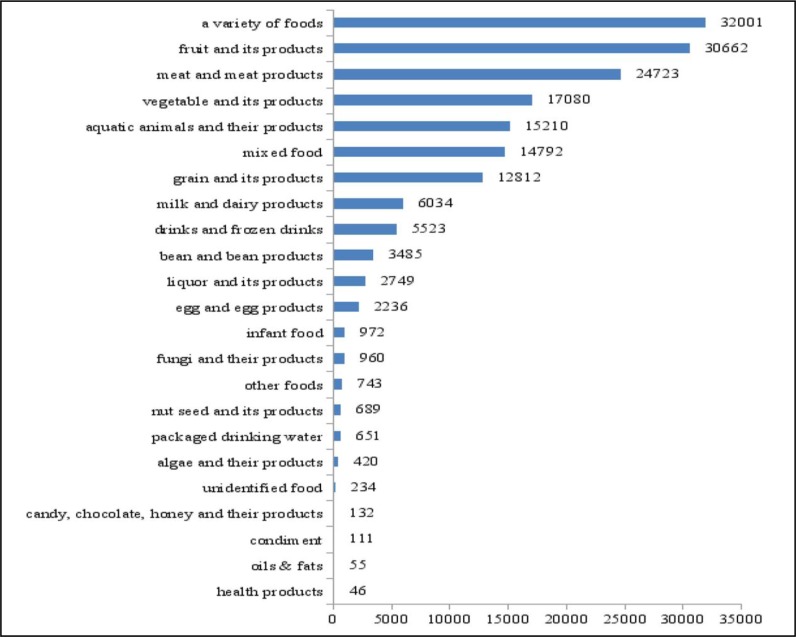
Number of surveillance cases of twenty-three categories, Shandong, 2016 – 2017. *Some cases have 2 or more exposed foods before the onset of the disease

The most frequently reported exposed food was a variety of foods (meaning more than two kinds of food), 32001 cases were reported. The second frequently reported exposed food was fruit and its products (including preserved fruit and preserves), causing more than 30000 cases. Other common exposure foods include meat and meat products, vegetable and its products, aquatic animals and their products, mixed foods and grain and its products (including starch sugar, roasting and all kinds of staple food), causing 24723 cases, 17080 cases, 15210 cases, 14792 cases and 12812 cases respectively (Fig. 2).

### Incidence of foodborne disease

The 150202 cases reported corresponded to a two-year average incidence rate of 75.78 cases per 100,000 population. The two-year average sex-specific incidence rate was much higher for females compared to males (78.23 vs. 74.69 cases per 100,000 population).

The highest two-year average incidence rates of foodborne diseases were reported in Dongying (117.11 cases per 100,000 persons) and Taian (115.52 cases per 100,000 persons).

In contrast, the lowest two-year average incidence rates of foodborne disease were observed in Qingdao (43.16 cases per 100,000 persons) and Liaocheng (47.54 cases per 100,000 persons) (Table 1).

**Table 1:** The incidence rate of different regions for cases reported, Shandong, 2016 to 2017

***Region***	***Total year-end population of 2015 (10000 persons)***	***The number of cases reported in 2016***	***The incidence rate of 2016 (100000 persons)***	***Total year-end population of 2016 (10000 persons)***	***The number of cases reported in 2017***	***The incidence rate of 2017 (100000 persons)***	***Two-year average rates (100000 persons)***
Jinan	713.20	3018	42.32	723.31	5127	70.88	56.60
Qingdao	909.70	3489	38.35	920.40	4415	47.97	43.16
Zibo	464.20	3300	71.09	468.69	6042	128.91	100.00
Zaozhuang	387.80	3036	78.29	391.56	3590	91.68	84.99
Dongying	211.06	1832	86.80	213.21	3143	147.41	117.11
Yantai	701.41	4938	70.40	706.40	10034	142.04	106.22
Weifang	927.72	3034	32.70	935.70	7248	77.46	55.08
Jining	829.92	6524	78.61	835.44	11227	134.38	106.50
Tai’an	560.08	4072	72.70	563.74	8926	158.34	115.52
Weihai	280.53	2604	92.82	281.93	3409	120.92	106.87
Rizhao	288.00	1741	60.45	290.11	2268	78.18	69.32
Laiwu	135.16	696	51.49	137.58	1275	92.67	72.08
Linyi	1031.16	5339	51.78	1044.30	7933	75.96	63.87
Dezhou	574.23	2069	36.03	579.23	3551	61.31	48.67
Liaocheng	597.06	2048	34.30	603.68	3669	60.78	47.54
Binzhou	385.90	1489	38.59	389.10	3367	86.53	62.56
Heze	850.03	5534	65.10	862.26	10215	118.47	91.79
Total	9847.16	54763	55.61	9946.64	95439	95.95	75.78

Overall, the highest two-year average incidence rate of foodborne disease was reported among children under 15 yr of age (90.49 cases per 100,000 persons) and the lowest was reported among persons no less than 65 yr of age (64.59 cases per 100,000 persons). There’s an age-specific trend were observed for the cases reported (Chi-Square for linear trend, χ^2^=4.39, *P*=0.036<0.05) (Table 2).

**Table 2:** The incidence rate of foodborne disease by age groups for cases reported, Shandong, 2016 to 2017

***Age groups***	***The number of cases reported in 2016***	***Total year-end population of 2015***	***The incidence rate of 2016 (100000 persons)***	***The number of cases reported in 2017***	***Total year-end population of 2016***	***The incidence rate of 2017 (100000 persons)***	***Total***	***Two-year average rates (100000 persons)***
≤14	9686	1634.62	59.26	19856	1631.24	121.72	29542	90.49
15-64	39377	7011.17	56.16	64854	7002.43	92.62	10423	74.39
							1	
≥65	5700	1201.35	47.45	10729	1312.95	81.72	16429	64.59
Total	54763	9847.16	55.61	95439	9946.64	95.95	15020	75.78
							2	

## Discussion

According to our analysis, private home was the most common exposure setting. However, further analysis of the source of food found that 1/3 of the cases reported in the private home were purchased from catering facility. In fact, catering facility with at least 64691 cases (43.1%) in our analysis. In spite of China and the United States have different categories of exposures, this proportion is close to the latest US report. According to ‘Surveillance for Foodborne Disease Out-breaks United States, 2014: Annual Report’ from the US CDC, 44.0% of foodborne disease outbreak cases reported restaurant as the location where food was prepared (12). Most of the cases are related to food prepared in a restaurant (13, 14).

Analysis of exposed foods of reported cases in Shandong, a variety of foods (meaning more than two kinds of food) were the most commonly reported classification. That means people eat more and more diverse foods, on the other hand, that means the category of exposed foods in China’s foodborne disease surveillance system is not specified in enough detail. Compared with western developed countries, China’s foodborne disease surveillance system still has many limitations and remains in the early stage of development in a stepwise fashion. Such as food categories implicated in foodborne disease outbreaks were 24 categories in the USA (14), no “a variety of foods”. Fruit and its products (including preserved fruit and preserves) was the second frequently reported exposed food, meat and meat products was only third in our analysis, this result is similar to that of other provinces in China (15, 16), however, the major proportion of cases of foodborne illness in western developed countries are associated with foods of animal origin (17). In the US and the EU, leafy greens, tomatoes, melons, legumes and grains were major food types in non-animal origin of foods that cause foodborne diseases (18, 19), and our analysis also shows that vegetable and its products is a very important food category of exposed food.

The assessment of the burden of acute gastroenteritis in the population is a central issue of the burden of foodborne diseases. To some extent, the assessment of foodborne diseases can be regarded as an assessment of foodborne acute gastroenteritis. The cross-sectional survey on acute gastroenteritis in the population of 6 provinces (it has been mentioned in the introduction) showed that the weighted monthly prevalence rate of acute gastroenteritis in Jiangsu was 4.7% and the incidence was 0.63 times per year (20), and the weighted monthly prevalence rate of acute gastroenteritis in Hangzhou-Jiaxing-Huzhou area of Zhejiang was 7.0% from Jul to Sep (21). According to the survey data of acute gastroenteritis in 6 provinces, the weighted monthly prevalence of acute gastroenteritis was 4.2% and the incidence was 0.56 times per man-year (6). The two-year average incidence rate of reported cases in Shandong province was 75.78 cases per 100,000 population, whatever the incidence or prevalence rate is far below the above value. In addition to the obvious regional differences in climate, environmental conditions and population subpopulations, factors that could explain this include using traditional monitoring techniques and the effectiveness of the reporting system itself. Using traditional monitoring techniques, the cases of foodborne disease often fail to be reported, because traditional monitoring techniques can only find cases who seek for medical advice. The ability of syndromic surveillance system mainly depends on the population dispersion of those affected, the data sources and syndrome definitions used and the healthcare provider’s ability to detect and report unusual cases (22). It is better to use the probability distribution to estimate the food-borne ratio of acute gastroenteritis to illustrate the uncertainty of the assessment. In recent years, western developed countries (such as the United States, Australia, and so on) have taken into account uncertainty in assessing the foodborne proportions of acute gastroenteritis (5, 7, 23).

### Limitations of this study

There were several limitations of the data used for this study. First, insufficient information on food exposure information, many cases of eating at home have not filled in the specific source of food. Leading to inaccurate exposure settings. Second, exposed food is not specified in enough detail, the category of “a variety of foods” masks the true category information of food. Third, recall bias most likely occurred because most of the cases go to the hospital from 2 to 3 d after their onset of symptoms. The ability of cases to recall all relevant exposures after this period of time undoubtedly presented challenges. Fourth, the association between exposure and illness was not usually corroborated by laboratory analyses of clinical specimens and/or environmental samples, or other analytic studies. The reported exposures do not necessarily represent a causal relationship with illness. Fifth, the main complaint or suspicion of infectious cases caused by food cannot be completely excluded from other diseases. Finally, the exposures identified were based on the case investigator’s best assessment of the potential source(s) of the case’s illness.

## Conclusion

Using case monitoring data of Shandong Province to assess the burden of foodborne disease, whatever the incidence or prevalence rate is far below the cross-sectional survey data value (75.78 cases per 100,000 population vs. 0.56 times per man-year, 4.2%). Even though, using case monitoring data is still able to estimate at least 80 thousand cases of foodborne disease in Shandong each year. If using the data value of the cross-sectional survey, a preliminary estimate of 14 million cases of foodborne disease in Shandong province each year. There-from, the burden of foodborne disease in Shandong is very heavy. To obtain accurate data of foodborne diseases in Shandong, future studies should focus on cross-sectional and cohort studies to facilitate the assessment of the distribution and burden of foodborne disease of the population. It is also possible to consider strengthening the burden of foodborne diseases in foodborne disease surveillance, but considering the status of foodborne disease surveillance, this will be an arduous process.

## Ethical considerations

Ethical issues (Including plagiarism, informed consent, misconduct, data fabrication and/or falsification, double publication and/or submission, redundancy, etc.) have been completely observed by the authors.
